# Supplementary material for: Synthetic Flavonoid BrCl-Flav—An Alternative Solution to Combat ESKAPE Pathogens
Source: Antibiotics (Basel). 2022 Oct 11;11(10):1389. doi: 10.3390/antibiotics11101389 (PMC9598271; doi:10.3390/antibiotics11101389)
Supplement: Supplementary file 1 [file antibiotics-11-01389-s001.zip › antibiotics-1964519-supplementary.pdf]

Supplementary Materials

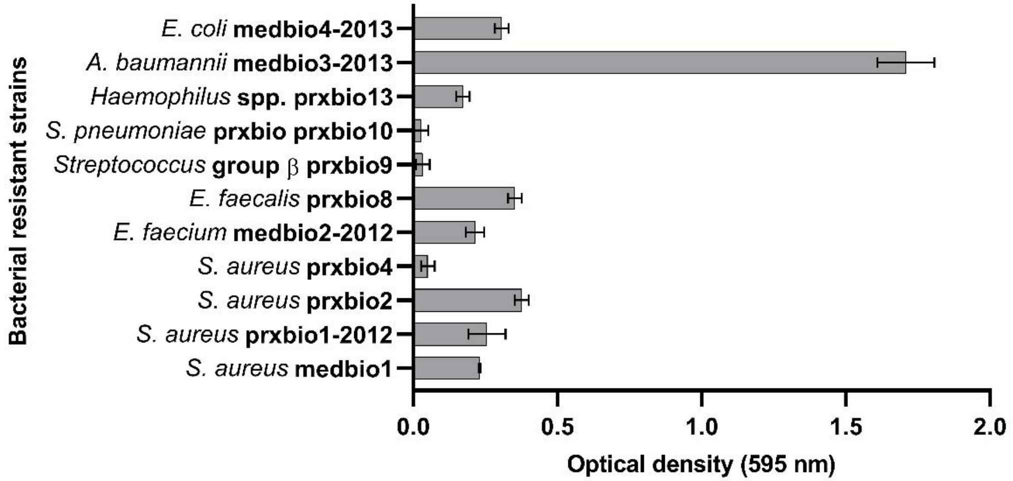

Figure S1. Biofilm-forming capacity of different clinical isolates. Bars indicate SEM.

Table S1. Antibiotic resistance profile of the tested clinical isolates

**Bacterial strains**	**Antibiotics**																						
	Amoxicillin/Clavulanic acid	Ampicillin	Ampicillin/Sulbactam	Cefazolin	Cefotaxime	Ceftazidime	Cefoxitin	Ciprofloxacin	Clindamycin	Chloramphenicol	Erythromycin	Fosfomycin	Gentamicin	Imipenem	Levofloxacin	Moxifloxacin	Nitrofurantoin	Oxacillin	Penicillin	Piperacillin	Piperacillin/Tazobactam	Tetracycline	Trimethoprim/Sulfamethoxazol
Staphylococcus aureus medbio1-2012	-	-	-	-	-	-	R	-	R	S	R	-	-	-	-	R	-	-	R	-	-	R	-
S. aureus prxbio1	-	-	-	-	-	-	S	-	S	S	S	-	-	-	-	S	-	-	R	-	-	S	-
S. aureus prxbio2	-	-	-	-	-	-	R	-	R	S	R	-	-	-	-	-	-	-	-	-	-	R	-
S. aureus prxbio3	-	-	-	-	-	-	S	S	S	-	R	-	S	-	S	-	-	-	-	-	-	-	S
S. aureus prxbio4	-	-	-	-	-	-	R	-	S	S	R	-	-	-	-	S	-	-	-	-	-	S	-
S. aureus prxbio5	-	-	-	-	-	-	R	R	S	S	R	-	S	-	-	-	-	-	-	-	-	R	-
S. aureus prxbio6	-	-	-	-	-	-	R	S	S	S	R	-	R	-	-	-	-	-	-	-	-	R	-
S. aureus prxbio7	-	-	-	-	-	-	S	-	S	S	R	-	-	-	-	S	-	-	-	-	-	R	-
Streptococcus spp. prxbio9	-	S	-	-	-	-	-	-	R	S	R	-	-	-	S	-	-	-	S	-	-	R	-
S. pneumoniae prxbio10	-	-	-	-		-	-	-	R	R	R	-	-	-	-	S	-	R	-	-	-	R	-

Enterococcus faecium medbio2-2012	-	R	-	-	-	-	-	R	-	-	-	S	R	-	R	-	R	-	R	-	-	-	-
E. faecalis prxbio8	-	S	-	-	-	-	-	S	-	S	-	-	R	-	S	-	-	-	-	-	-	R	-
Acinetobacter baumannii medbio3-2013	-	-	S	-	I	R	-	S	-	-	-	-	S	S	-	-	-	-	-	S	-	-	S
Escherichia coli medbio4-2013	S	R	-	R	R	-	-	R	-	-	-	-	S	-	-	-	S	-	-	-	-	-	R
Enterobacter cloacae medbio5-2013	R	R	-	R	R	-	-	S	-	-	-	-	S	-	-	-	R	-	-	-	-	-	S
Klebsiella pneumoniae medbio6-2013	R	R	-	R	R	-	-	R	-	-	-	-	R	-	-	-	R	-	-	-	-	-	S
K. pneumoniae prxbio11	R	-	S		S	S	-	S	-	-	-	-	-	-	S	-	-	-	-	S	-	-	S
K. pneumoniae prxbio12	S	R	-	R	R	-	-	R	-	-	-	-	S	-	-	-	R	-	-	-	-	-	S
Pseudomonas aeruginosa medbio7-2013	-	-	-	-	-	R	-	S	-	-	-	-	S	S	S	-	-	-	-	S	S	-	-
Salmonella enterica medbio8-2013	-	S	-	-	-	-	-	S	-	-	-	-	-	-	-	-	-	-	-	-	-	-	S
Haemophillus spp prxbio13	S	-	-	-	S	-	-	S	-	S	-	-	-	-	-	S	-	-	-	-	-	R	-

R = resistant; I = intermediate; S= sensitive
